# Depth variation in benthic community response to repeated marine heatwaves on remote Central Indian Ocean reefs

**DOI:** 10.1098/rsos.231246

**Published:** 2024-03-27

**Authors:** Sivajyodee Sannassy Pilly, Ronan C. Roche, Laura E. Richardson, John R. Turner

**Affiliations:** ^1^ School of Ocean Sciences, Bangor University, Bangor LL59 5AB, UK

**Keywords:** climate-induced thermal stress, marine heatwaves, depth zonation, benthic communities, shallow coral reefs, remote reef systems

## Abstract

Coral reefs are increasingly impacted by climate-induced warming events. However, there is limited empirical evidence on the variation in the response of shallow coral reef communities to thermal stress across depths. Here, we assess depth-dependent changes in coral reef benthic communities following successive marine heatwaves from 2015 to 2017 across a 5–25 m depth gradient in the remote Chagos Archipelago, Central Indian Ocean. Our analyses show an overall decline in hard and soft coral cover and an increase in crustose coralline algae, sponge and reef pavement following successive marine heatwaves on the remote reef system. Our findings indicate that the changes in benthic communities in response to elevated seawater temperatures varied across depths. We found greater changes in benthic group cover at shallow depths (5–15 m) compared with deeper zones (15–25 m). The loss of hard coral cover was better predicted by initial thermal stress, while the loss of soft coral was associated with repeated thermal stress following successive warming events. Our study shows that benthic communities extending to 25 m depth were impacted by successive marine heatwaves, supporting concerns about the resilience of shallow coral reef communities to increasingly severe climate-driven warming events.

## 1. Introduction

Climate-induced thermal stress is one of the main drivers of change in coral reef benthic communities [1–3]. However, the degree to which disturbance events affect ecological communities is not uniform across space and varies along environmental gradients [4–6]. Coral reefs show significant spatial and temporal heterogeneity in community structure post-disturbance [7–9]. While most assessments of the extent of coral bleaching and consequent degradation and succession processes are carried out in shallow depths (2–10 m) [7,10–13], the response of shallow benthic communities to thermal stress over depth ranges extending to 30 m is poorly described [9,14]. As light, temperature [15] and waves decrease with increasing depth, and the availability of organic resources increases at depths [16,17], it is expected that the response of benthic communities to thermal stress will dampen [18]. However, contrary to the deep reef refuge hypothesis, which suggests that reefs at greater depths could escape the effects of climate-induced bleaching events [18,19], increasing evidence of bleaching and mortality within shallow (2–27 m) and mesophotic depth zones (≥ 30 m) [8,14,20–27] underlines the necessity to assess communities at larger depth gradients. Additionally, the limited overlap of species between shallow and deep reefs [28,29] and significant genetic divergence between species across depths indicate that deeper populations may not always provide viable propagules to repopulate shallow reefs [30,31]. These observations have important implications for coral reef assessments that evaluate the ecological response of shallow reef organisms to increasingly frequent thermal stress events, which are predicted to occur annually within the twenty-first century [32].

In 2014–2017, the world observed the first back-to-back coral bleaching events that were documented over 3 years [33–35]. The recurring heatwaves caused coral bleaching on 80% of coral reefs globally and the mass mortality of corals [1,11,35,36]. Thermal anomalies such as the 2014–2017 warming events have raised global concern about the resilience and persistence of tropical reef systems [1,35]. Chronic disturbances can impede recovery and induce significant degradation in communities [37]. As the increased intensity and frequency of ocean-warming events [38–40] shorten the recovery window for benthic communities [41–43], they can lead to potentially irreversible ecological changes [44,45].

Large-scale bleaching-induced coral mortality on coral reefs alters the dynamic interactions between benthic groups, causing significant shifts in community assemblages [46,47]. While the loss of hard corals can cause bare reef pavement to dominate the substrate [48,49], ecological regime shifts on coral reefs can generate non-coral-dominated states primarily characterized by benthic taxa such as algae (including crustose coralline algae (CCA), turf and macroalgae), soft corals and sponges [47,50–54]. Opportunistic and faster growing in nature, these alternative benthic groups competitively replace hard corals, which can lead to a loss of complexity [55,56], reduce reef carbonate budgets [57,58] and alter reef-associated fish communities [59,60], resulting in a decline in provision of important ecosystem services [61,62]. In some instances, coral community composition is altered despite recovering to pre-disturbance coral abundance [7,63,64].

While climate-driven community changes may result in degraded and less resilient reefs [23,65], the depth variability in changes in benthic community assemblages is poorly understood. Coral reef benthic communities are highly heterogeneous [66,67] and naturally show diverse vertical zonation patterns [28,68,69] as a result of the interaction between biophysical processes that concomitantly vary across depths [17,70]. During a marine heatwave, modifications in physiologically important factors such as the combination of sustained elevated seawater temperatures and high solar radiation are often the main causes of bleaching and degradation on reefs [18,71,72]. Additionally, the proportion of change in benthic communities following a marine heatwave may be due to the assemblage composition [11]. Studies show that coral species have different thermal tolerance [73–75], and the susceptibility of species within the same genus to climate-induced bleaching can vary significantly across depths [14]. In addition, imports of nutrient-rich and cold water from deep-water upwelling and internal waves onto shallow reef systems can confer resilience to change in ecological communities during ocean-warming events [76,77]. High spatial variation is therefore expected to occur in the response of benthic lifeforms to thermal stress within the water column [14,78,79].

Where post-bleaching coral reef assessments incorporate multiple depth zones, they can estimate depth-dependent mortality, identify surviving populations with the potential to repopulate and provide a better understanding of the trajectory of community changes across the extent of shallow reef systems (0–30 m) [14,25,27,78,80]. Here, we use benthic community surveys from shallow forereefs of the remote Chagos Archipelago, Central Indian Ocean, before and after successive marine heatwaves in 2015–2017. We examine the effects of recurrent severe thermal stress on benthic communities across a depth gradient of 5–25 m. In 2015–2017, the marine heatwave caused severe coral bleaching and mortality in the Chagos Archipelago, with sustained declines in coral cover due to two consecutive years of elevated temperature stress [41]. Specifically, we assess the change in percentage cover of five broad benthic groups and examine spatial variation in the change in composition of those groups across depths at four atolls in the archipelago. We also examine differences between the initial and repeated effects of thermal stress on the observed changes in benthic composition.

## 2. Material and methods

### 2.1. Study sites

**Figure 1.**
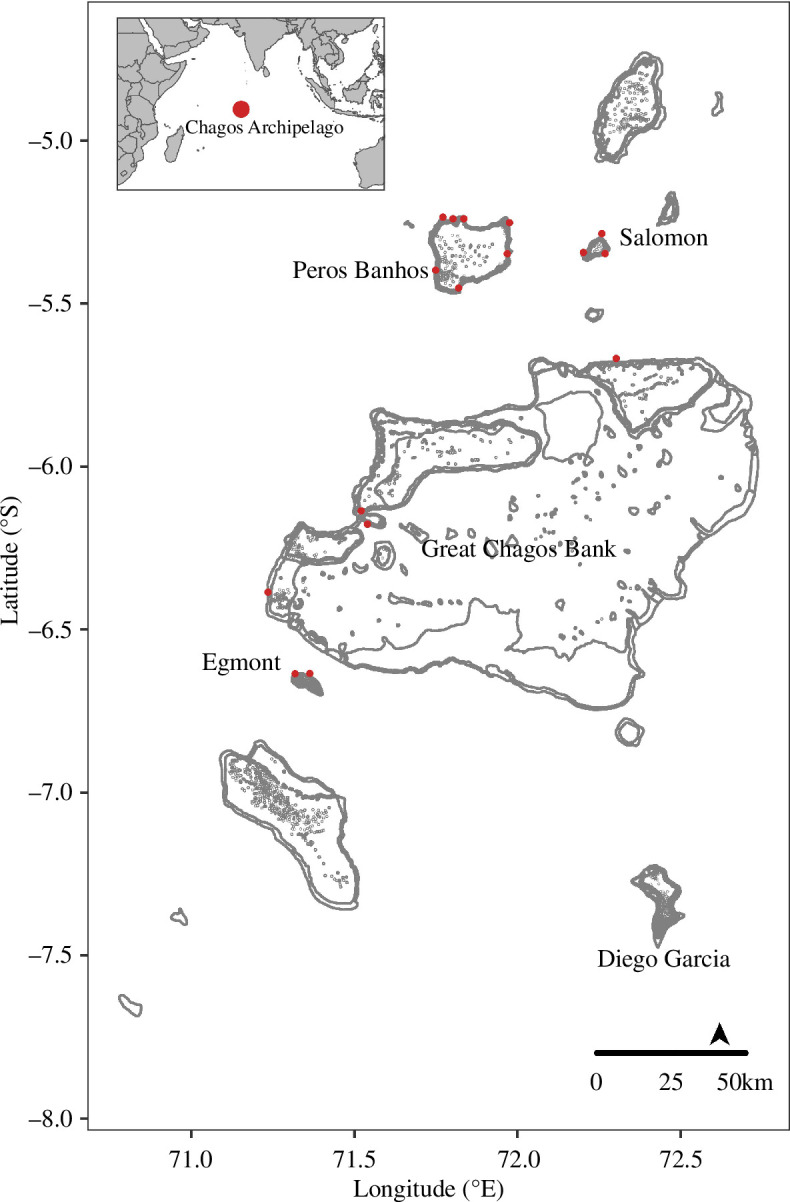
Map of sampled sites (red points) around surveyed atolls (in bold) in the Chagos Archipelago—northern atolls: Peros Banhos, Salomon and southern atolls: Great Chagos Bank and Egmont—see electronic supplementary material, table S1, for a list of sites and coordinates.

The Chagos Archipelago is a remote archipelago located in the Central Indian Ocean. Situated at the southern end of the Laccadives–Maldives–Chagos ridge, it covers a total area of 640 000 km^2^ and consists of five islanded atolls. The archipelago has been uninhabited since the early 1970s, except for the US military base on the southern atoll, Diego Garcia [81]. Benthic community data were collected with repeat sampling in March–April 2013–2014 (before) and 2018–2019 (after) following successive severe marine heatwaves in 2015–2017. The peaks of marine heatwave events occurred in May–June 2015 (4.2 ± 0.4–6.1 ± 0.3°C-weeks) and April–June 2016 (12.1 ± 0.1–15.8 ± 0.1°C-weeks) (electronic supplementary material, figure S1). Surveys in each period were carried out at 16 sites on forereef slopes across four atolls—Peros Banhos (PB), Salomon (SA), Great Chagos Bank (GCB) and Egmont (EG) (figure 1)—and conducted at four depth zones: 5–10, 10–15, 15–20 and 20–25 m. For ease of interpretation, shallower depth zones in the text refer to benthic groups occurring between 5–15 m and deeper zones to benthic groups occurring between 15–25 m.

### 2.2. Data collection and benthic community assessment

A benthic community assessment was carried out using 10 min continuous video swims conducted at a constant speed (0.1 m s^−1^) at each site and depth zone. Equipped with two spotlights and two red laser pointers set 10 cm apart, the camera set-up provided a scale of measurement of the benthos and adjustment for lower light levels at greater depth. During the survey, the camera was held approximately 0.5 m above the substrate and at a 45° angle to capture benthic organisms and substrate types under overhangs and canopies [82]. Each video was converted into a sequence of still images (25 frames per second in Pinnacle Studio, v. 22.2.0). Thirty of these images were selected for analysis using Matlab (R2018a.Ink; total across all sites, *n* = 5610). Selected images were separated by 80–100 frames in each sequence to avoid resampling the same area of reef.

Benthic composition within each image was quantified using Coral Point Count with Excel extensions [83]. To reduce observer bias, image analysis was equally distributed among S.S.P., R.C.R. and L.E.R. (10 images per person per site). The percentage cover of benthic organisms and other substrate types was quantified at 15 randomly allocated points on each image using a stratified random design [84]. The broad classification of benthic groups was adapted from Denis *et al*. [85] and the National Oceanic and Atmospheric Administration (NOAA) Coral Reef Information System [86]. This study examined five benthic groups that have been found to undergo shifts in dominance and contribute to habitat building on coral reefs following thermal stress events: hard coral, soft coral, sponge, CCA and reef pavement [48,87–90]. Despite being an important competitor to corals [47], macroalgae were not included, as we only found small changes in macroalgal cover (turf and fleshy macroalgae) following marine heatwaves (pre-heatwaves: 6.5 ± 0.2% and post-heatwaves: 6.0 ± 0.1%).

### 2.3. Estimating change in benthic groups following 2015–2017 bleaching events

The change in the benthic group cover which occurred following the 2015–2017 warming events was calculated using


$$\delta =\frac{\log\left(V_{f}\right)-\log\left(V_{0}\right)}{t},$$

where δ is the geometric logarithmic change in cover, *V*_*f*_ is the percentage cover of benthic groups collected at the end of the time series, i.e. 2018 or 2019, *V*_0_ is the percentage cover of benthic groups at the beginning of the time series, i.e. 2013 or 2014, and *t* is the duration of the time in years between benthic surveys, i.e. before and after the successive marine heatwaves [91]. This approach optimized the dataset by including surveyed sites that were monitored in different years before and after the heat stress event, i.e. to include pre-disturbance community data collected in 2013 or 2014 (hereafter pre-heatwaves), and post-disturbance community data sampled in 2018 or 2019 (hereafter post-heatwaves). The geometric logarithmic change in cover metric allows the quantification and comparison of nonlinear time series by taking into consideration the exponential decline and increase in cover of benthic communities over time (*sensu* [91]; e.g. [64,92]). Compared with other metrics that measure ecological change over time [92], the geometric logarithmic rate of change preserves proportionality of change in cover (e.g. a 50% to 5% loss is proportionately similar to a 10% to 1% loss) and is symmetrical, i.e. produces similar rates of change for declines and matching increases (e.g. a 50% to 5% loss and a 5% to 50% increase yield the same rates [91]). To summarize the geometric logarithmic change in cover metric, a negative value represents a loss in benthic group cover from pre- to post-heatwaves and a positive value indicates an increase in benthic group cover following thermal stress. Variation in the change in benthic group cover across depth zones was visualized using a forest plot (ggplot: ggplot2 package [93]). For ease of interpretation, the log change in cover was back-transformed to a percentage change in cover (electronic supplementary material, figure S2), using


$$i=\left(e^{\delta }-1\right)\times 100,$$

where *δ* is the geometric logarithmic change in cover, and *i* is the percentage change in cover.

### 2.4. Exposure to repeated thermal stress

To determine how benthic groups were affected by thermal stress across depth zones during the 2015–2017 marine heatwaves, maximum degree heat week (hereafter maxDHW) was used to quantify thermal anomalies from 2015 to 2017. DHW is a proxy for accumulated thermal stress, represented by a 1°C increase above the local mean climatic temperature over a 12-week period at a given pixel and expressed as degree Celsius weeks (°C-weeks); and maxDHW is the annual maximum accumulated thermal stress in a year. Using the NOAA Coral Reef Watch 5 km resolution product [94], the annual maxDHW between January 2015 and December 2017 was extracted for each study site (using R packages: ncdf4 [95], raster [96], rgdal [97] and sp [98]). A DHW threshold of 4°C-weeks may be indicative of significant coral bleaching, and a DHW value of 8°C-weeks is a signal for severe and widespread bleaching with likely mortality [11,99–102]. Local-scale hydrodynamics, e.g. upwelling and changes in mixed layer depth, can influence temperature regime across depth gradients [103,104]. However, in the absence of depth-specific temperature data from *in situ* loggers across all study sites, DHW that was derived from SST was used as a proxy of thermal stress experienced along the depth gradient at the study sites during the warming events (electronic supplementary material, figure S1). Here, initial thermal stress refers to maxDHW recorded in 2015 in the first year of the successive marine heatwaves (4.1–6.4°C-weeks) and repeated thermal stress refers to the cumulative maxDHW recorded in all years from 2015 to 2017 (17.1–21.1°C-weeks, electronic supplementary material, table S1). Cumulative maxDHW has been shown to be a good predictor of bleaching in the Western Indian Ocean [105] and therefore the change in benthic group cover following repeated marine heatwaves.

### 2.5. Data analysis

Non-metric multi-dimensional scaling (nMDS: vegan package [106]) analysis was used to visualize variation in benthic community composition across and within depth zones among atolls before and after the thermal stress. Using a Bray–Curtis distance of square-root transformed data [107], the nMDS was computed in three dimensions (*k* = 3) with a stress value of less than 0.1. A scree plot and a Shepard stress plot were used to assess the ordination stress and the correlation between the original dissimilarity matrix and the distances on the final nMDS plot. Vectors of the different benthic groups were fitted to show their correlations with how atolls and depth zones are clustered on the ordination plots (envfit: vegan package; with 9999 permutations).

A three-way nested permutational multivariate analysis of variance (PERMANOVA) was carried out to assess whether benthic community composition varied across depth zones (four levels; fixed factor) among atolls (four levels, fixed factor), and whether variation was observed before and after the successive heatwaves (two levels; fixed factor), including an interaction term between depth zone, atoll and heatwave period (i.e. pre- and post-heatwaves). Sites (random factors) were nested in atolls to account for and control for site-specific variation in benthic communities to isolate the effects of the fixed factors. Multivariate homogeneity tests identified whether there were differences in benthic community dispersion across depth zones before and after successive heatwaves (betadisper: vegan package, using Bray–Curtis distances between samples). The difference between the mean dispersion in benthic communities before and after the successive heatwaves across depth zones was tested using a permutation test (permutest: vegan package, with 9999 permutations). Where a significant interaction between depth zones, atolls and successive heatwaves was found, Bonferroni pairwise comparisons of group mean differences were also performed to contrast pre- and post-heatwave communities within depth zones and among atolls.

Bayesian hierarchical models were used to assess the effects of initial and repeated thermal stress on the change in cover of each benthic group (hard coral, soft coral, sponge, CCA and reef pavement) across depth zones and among atolls (brm: brms package [108]). A generalized linear mixed-effects framework was used to model change in cover (response variable), with a Gaussian distribution, as a function of depth zone, atolls and initial and repeated thermal stress (population-level effects), with an interaction between depth zone, atoll and each thermal stress variable. The site (group-level variable) was nested in the atoll to control for the spatial variation in the change in benthic groups at the site level. The models were run in Stan (brms package), using weakly informative priors for the regression parameters in the model (electronic supplementary material, table S2). Models were fitted with 2000 iterations across four chains using a Markov chain Monte Carlo (MCMC) algorithm. Excluding 1000 warm-up iterations per chain, all posterior sampling included 4000 draws to simulate the response variables. Predictors (initial and repeated thermal stress) were centred, and the convergence of the MCMC algorithm was visually assessed using traceplots. Effective and reliable sampling of the posterior distributions was assessed using Gelman–Rubin convergence R-hat values of less than 1.05 and a minimum effective sample size of less than 1000 for all parameters [109]. Posterior predictive checks were used to assess model fits (bayesplot package [110] and tidybayes package [111]). The influence of each predictor (depth zone, atoll, initial and repeated thermal stress) on the change in benthic group cover following the successive severe marine heatwaves was assessed using average marginal effects (emmeans, emtrend: emmeans package [112]). Uncertainty related to the models’ posterior estimates was interpreted with 50% and 95% credible intervals. Strong and weak evidence of change was interpreted when 95% and 50% of the intervals did not intercept zero, respectively [64,113]. All data analyses were performed using R 3.5.1 [114].

## 3. Results

### 3.1. Variation in benthic community composition after 2015–2017 marine heatwaves

**Figure 2.**
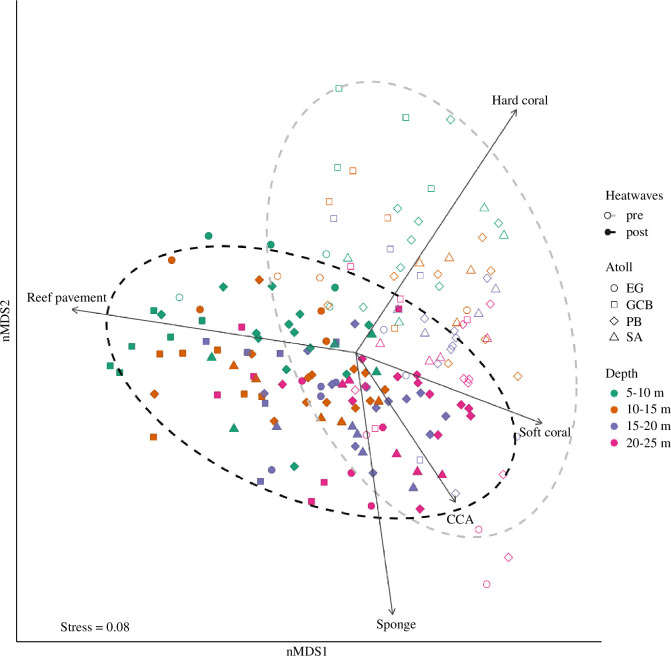
Non-metric multi-dimensional scaling plot of benthic groups from 16 sites in the Chagos Archipelago, showing clustering of communities in 2013–2014 (pre-heatwaves: unfilled shapes) and 2018–2019 (post-heatwaves: filled shapes) following 2015–2017 marine heatwaves across depth zones (green: 5–10 m, orange: 10–15 m, blue: 15–20 m and pink: 20–25 m) at atolls—Egmont (EG), Great Chagos Bank (GCB), Peros Banhos (PB) and Salomon (SA)—based on Bray–Curtis dissimilarities of square-root transformed data. Ellipses represent the dispersion of pre (grey lines) and post (black lines) marine heatwave communities from centroids at 95% confidence intervals. Vectors show benthic groups that significantly contributed to the patterns on the ordination; arrows show the direction of the gradient; and the length of the vectors is proportional to the correlations between the benthic group and the ordination. Depth-dependent and atoll-dependent variation in benthic communities can be viewed in electronic supplementary material, figure S3.

Multivariate analyses showed no interaction effect between depth and atoll on benthic communities (figure 2, table 1). These findings indicate that there was no difference in benthic communities before and after the marine heatwaves among atolls across depth zones. However, significant depth-dependent and atoll-dependent variations in benthic community composition before and after the successive heatwaves were recorded across all 16 study sites (figure 2, table 1). Pairwise comparisons showed significant differences in benthic communities before and after the successive heatwaves across all depth zones, except at 20–25 m, and across all atolls, except at Egmont (electronic supplementary material, figure S3 and table S3). Dispersion analyses revealed similar dispersion means for benthic communities pre- and post-heatwaves across all depth zones and atolls (table 1).

**Table 1.** Variation in the benthic community before and after the 2015–2017 marine heatwaves using permutational analysis of variance (PERMANOVA) and dispersions tests across depth zones (5–10, 10–15, 15–20 and 20–25 m) among atolls (Egmont, Great Chagos Bank, Peros Banhos and Salomon).

	PERMANOVA			dispersion		
	d.f.	pseudo-*f*	*p*‐value	d.f.	pseudo-*f*	*p*‐value
Heatwaves × Depth × Atoll	9,127	0.72	0.8274			
Heatwaves × Depth	3,186	5.98	0.0001	7,120	0.4958	0.8361
Heatwaves × Atoll	3,186	5.64	0.0001	7,120	1.0796	0.3807
Depth x Atoll	9,186	1.5796	0.0607			

*Notes:* d.f., degrees of freedom

### 3.2. Depth-dependent temporal change in benthic composition

#### 3.2.1. Decline in hard and soft coral cover

**Figure 3.**
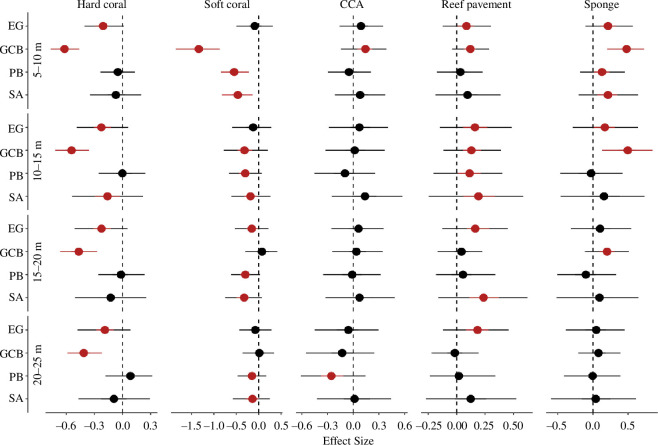
Standardised effects of depth zones (from multi-level Bayesian models) on change in benthic group cover following the 2015–2017 marine heatwaves at atolls: Egmont (EG), Great Chagos Bank (GCB), Peros Banhos (PB) and Salomon (SA). Points indicate median posterior estimates, and bars represent 50% and 95% credible intervals. Strong and weak evidence of change in benthic cover is interpreted when 95% (red lines) and 50% (black and red lines) of the intervals do not intercept zero, respectively. Non-significant effects are shown by black lines.

Our results revealed an overall decline in hard coral (−14.1 ± 1.5%) and soft coral cover (−23.1 ± 3.1%), which varied across depth zones and atolls following the 2015–2017 marine heatwaves. Hard coral cover declined in all depth zones (5–25 m) at Great Chagos Bank compared with Egmont, where a small gain was observed (figure 3, electronic supplementary material, table S4). The loss in hard coral cover after bleaching was greatest at 5–10 m at Great Chagos Bank (figure 3, electronic supplementary material, table S4) and declined with increasing depth (figure 3, electronic supplementary material, table S4). At three atolls (Great Chagos Bank, Peros Banhos and Salomon) soft coral cover declines were greatest at 5–10 m (figure 3, electronic supplementary material, table S4). Soft coral cover loss became less pronounced with increasing depth at all atolls; however, marginal declines were still observed at 15–20 m at Egmont, Peros Banhos and Salomon (figure 3, electronic supplementary material, table S4).

#### 3.2.2. Increase in crustose coralline algae, reef pavement and sponge cover

There was an overall 6.6 ± 1.7% increase in CCA cover following successive heatwaves. There was no evidence of a depth effect on the change in CCA cover at any atoll, except for an increase in CCA at Great Chagos Bank at 5–10 m and a loss in CCA cover at 20–25 m at Peros Banhos (figure 3, electronic supplementary material, table S4).

Reef pavement increased by 13.4 ± 1.7% post-heatwaves. The increase in reef pavement at 5–10 m was more pronounced at Egmont and Great Chagos Bank (figure 3, electronic supplementary material, table S4). All atolls showed an increase in reef pavement at 10–15 m (figure 3, electronic supplementary material, table S4). Reef pavement marginally increased at deeper zones (15–20 m and 20–25 m) at Egmont and Salomon (figure 3, electronic supplementary material, table S4).

There was an increase in sponge cover (24.1 ± 7.6%) following successive heatwaves. A small increase in sponge cover was observed at all atolls at 5–10 m and at 10–15 m at Egmont and Great Chagos Bank (figure 3, electronic supplementary material, table S4). Sponge cover marginally increased at 15–20 m at Great Chagos Bank. However, there was no change in sponge cover at 20–25 m at any of the atolls (figure 3, electronic supplementary material, table S4).

### 3.3. Effects of thermal stress on change in benthic communities across depth zones

**Figure 4.**
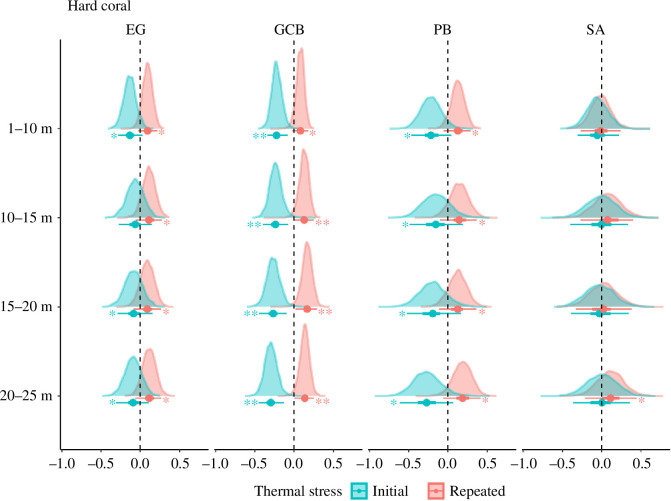
Posterior distributions of the standardised effects of initial and repeated thermal stress (from multi-level Bayesian models) on the change in hard coral cover following 2015–2017 marine heatwaves across depth zones: 5–10, 10–15, 15–20 and 20–25 m at atolls: Egmont (EG), Great Chagos Bank (GCB), Peros Banhos (PB) and Salomon (SA). Points indicate median estimates, and bars represent 50% and 95% credible intervals. Strong and weak evidence of change in benthic cover is interpreted when 95% (indicated by **) and 50% (indicated by *) of the intervals do not intercept zero, respectively.

Variable effects of initial and repeated thermal stress were observed on the change in benthic groups across depth zones and among atolls. The loss in hard coral cover at all depths was driven by initial thermal stress, with consistent negative effects on the decline in hard coral cover across depth zones (figure 4, electronic supplementary material, table S5). Stronger evidence of hard coral cover loss due to initial thermal stress was shown at Great Chagos Bank across all depth zones, with less pronounced effects at Peros Banhos and Egmont (figure 4, electronic supplementary material, table S5). Repeated thermal stress had a marginal effect on the decline in hard coral cover across all depth zones. These effects were more pronounced at Great Chagos Bank in deeper zones (10–25 m) (figure 4, electronic supplementary material, table S5).

Unlike hard coral, changes in soft coral, CCA, reef pavement and sponge cover following successive marine heatwaves were weakly and variably affected by initial and repeated thermal stress across depth zones and among atolls. Great Chagos Bank was one of the atolls where both initial and repeated thermal stress marginally influenced the loss in soft coral cover across depth (electronic supplementary material, figure S4 and table S5). Both initial and repeated thermal stress marginally decreased soft coral cover at 20–25 m at Egmont. At Salomon, soft coral cover marginally declined due to initial thermal stress at 20–25 m (electronic supplementary material, figure S4 and table S5).

Initial thermal stress marginally increased CCA cover at 5–10 m at Great Chagos Bank (electronic supplementary material, figure S5 and table S5). There was no further evidence of an effect of initial thermal stress at other depth zones and atolls (electronic supplementary material, figure S5 and table S5). Repeated thermal stress marginally increased CCA cover at Peros Banhos at shallower depth zones (5–10 and 10–15 m). The effects of repeated thermal stress on the change in CCA cover were divergent at 20–25 m, with a small increase at Great Chagos Bank and a small decrease at Peros Banhos (electronic supplementary material, figure S5 and table S5).

There was no evidence of an effect of initial thermal stress on the increase in reef pavement at 5–10 m at any atoll. Repeated thermal stress slightly increased reef pavement at 5–10 m at Great Chagos Bank and Salomon (electronic supplementary material, figure S6 and table S5). Both initial and repeated thermal stress marginally increased reef pavement at deeper zones (10–15, 15–20 and 20–25 m) at Egmont, Great Chagos Bank and Salomon (electronic supplementary material, figure S6 and table S5).

Initial thermal stress marginally increased sponge cover at shallower depth zones (5–10, 10–15 m) at Great Chagos Bank and at deeper zones (20–25 m) at Peros Banhos. A slight increase in sponge cover was observed due to repeated thermal stress at mid-depths (10–15, 15–20 m) at Egmont, Great Chagos Bank and Peros Banhos (electronic supplementary material, figure S7 and table S5).

## 4. Discussion

It is evident that intense ocean-warming events such as the successive heatwaves in 2014–2017 [36,115] are changing coral-dominated reefs to alternative configurations, with severe impacts on ecosystem functioning [11]. However, there is limited understanding of the changes that occur in benthic communities from recurring warming events at depths greater than 10 m [1,60,101,116]. We observed changes in benthic communities on the remote Central Indian Ocean reefs of the Chagos Archipelago following successive heatwaves in 2015–2017 at depths down to 25 m. Our findings indicate greater changes in benthic group cover at shallower depths (5–15 m) relative to deeper zones (15–25 m). However, the changes in benthic communities in response to elevated seawater temperatures were variable at different depths.

### 4.1. Variation in ecological response to thermal stress across depth

Changes in communities occur as a result of the complex interactions between their habitat, physiological traits and the nature of disturbance events [117]. Here, we show that the change in benthic communities decreased with increasing depth. This pattern [14,78] may relate to biophysical forcing, such as light, temperature, salinity [15,118,119] and wave and current regimes [16,120] that simultaneously vary across depth.

During a global warming event, compositional changes observed in benthic communities occur as a result of thermal stress-related coral bleaching, which is caused by increased seawater temperature and light irradiance. As light intensity and temperature naturally decline with increasing depth [15], lower levels of bleaching and mortality are frequently observed at depth [8,14,24,25,27], which could account for the lower change in soft and hard coral cover at 15–25 m compared with the shallower communities at 5–15 m. In addition to the combination of the natural attenuation of light and temperature with increasing depth, the presence of internal waves may explain the smaller change in benthic community cover at deeper zones relative to shallower parts of the reefs. Significant water temperature fluctuations with increasing depth suggest strong internal wave activity in the Chagos Archipelago [121]. Internal waves which drive deep-water upwelling can significantly decrease thermal stress and mitigate the response of benthic organisms to bleaching events at depth [122]. By cooling temperatures and bringing in allochthonous nutrients from the deep, upwelling can increase productivity in shallow reef systems [120]. When autotrophy is compromised during a warming event [123], upwelling can promote heterotrophy and the survival of mixotrophic organisms such as hard and soft corals [17,77,124,125].

Despite the variation in the change of benthic groups across depths, we observe a consistent pattern of loss in hard coral and soft coral cover, followed by an increase in CCA, reef pavement and sponge cover across all depth zones. The increase in sponge and CCA cover can also be associated with the increase in the proportion of reef pavement after the marine heatwaves. Mass bleaching events have been shown to induce widespread mortality in hard and soft coral communities [40,126] creating vacant space [127]. Recent studies in the Chagos Archipelago have shown a higher proportion of reef pavement and boring sponge cover on shallow exposed sites after the 2015–2017 marine heatwaves compared with more sheltered sites [48]. Higher wave energy at shallow depths can increase the susceptibility of benthic groups such as dead hard and soft corals to physical damage and dislodgement [128], resulting in vacant bare reef pavement and promoting the growth of rapidly colonizing and wave-tolerant organisms like sponge and CCA [129,130].

### 4.2. Variation in ecological response to initial and repeated thermal stress

#### 4.2.1. Change in hard coral cover following bleaching events

There were variable and modest responses in benthic communities to initial and repeated thermal stress across depth zones. Recent studies show similar trends of little to no significant interaction between depth and thermal stress in explaining post-heatwave trajectories in benthic communities across depth gradients [8,26,131]. Among all surveyed benthic groups, only hard coral cover had a consistent negative effect of initial thermal stress across all depth zones. This suggests that the initial effect of thermal stress in 2015 had the largest influence on the observed decline in hard coral cover, potentially due to mortality of coral assemblages with low tolerance to thermal stress [101,117]. In contrast, the cumulative effect of repeated thermal stress from 2015 to 2017 was associated with a smaller decline in hard coral cover. Previous studies in Chagos support these findings with comparable patterns reporting higher coral bleaching and mortality in 2015 despite higher exposure to thermal stress in 2016 [41]. Similar trends of lower bleaching and mortality rates in the second year of consecutive coral bleaching events were reported on the Great Barrier Reef [116] and in the Coral Sea [101], even though higher thermal stress was recorded in the second year.

There are several mechanisms which could drive successive bleaching events resulting in lower coral mortality. Over the duration of a warming event, less susceptible hard corals can resist, adapt and recover from thermal stress [132,133]. For example, exposure to the initial warming event in 2015 may have created thermal preconditioning for the remaining live hard coral community to develop resistance to subsequent bleaching [134,135]. In addition, natural association with [136,137] or shifting to thermally tolerant endosymbionts during stress [138,139] can decrease bleaching susceptibility and lower coral cover decline during subsequent warming events.

#### 4.2.2. Change in other benthic groups following bleaching events

Unlike the hard coral community, the remaining benthic groups showed weak and variable responses to both initial and repeated thermal stress across depth zones. For example, repeated thermal stress increased soft coral cover loss at 20–25 m, whereas no effects were observed on change in soft coral cover at shallower depths. Studies which have looked at the impact of recurring thermal stress on soft coral communities in the Western and Central Pacific also show similar trends of loss related to repeated bleaching and eventual die-offs [88,126]. The impact of thermal stress at 20–25 m may relate to lower thermal variability at depths making deeper soft coral communities less resilient to chronic warming [88]. In addition, soft coral communities at depth could be made up of more vulnerable populations like *Sarcophyton* colonies, which are known to degrade completely after recurring bleaching events, compared with *Lobophytum* and *Sinularia,* which are more resistant to bleaching [88,126].

Both initial and repeated thermal stress decreased the recovery in sponge cover following the 2015–2017 bleaching event. This effect was most pronounced at the shallowest depths (5–10 m). The increase in sponge cover that we found is probably driven by an increase in encrusting boring sponges [90,140], also observed following the major 1998 bleaching event in the Chagos Archipelago [141]. These bio-eroding sponges can host *Symbiodinium* spp., which are resistant to bleaching [142]. This association with thermally tolerant holobionts helps the sponges spread rapidly on stressed and dead corals during warming events [143].

CCA was the benthic group least influenced by initial and repeated thermal stress. Repeated thermal stress marginally reduced the recovery of CCA cover at one atoll (Peros Banhos), and minimal to no effect of initial thermal stress was observed on the increase in CCA cover. Recent studies show that CCA has high thresholds for elevated water temperatures [89]. While an acute thermal stress event can significantly reduce photosynthetic rates in CCA, it also has the potential to acclimatize to chronically elevated water temperatures and maintain photosynthesis and calcification [144]. In addition to being highly tolerant to heat stress, the increase in CCA cover may relate to their high dispersal rates and ability to spread rapidly on various substrates such as available bare reef substrate, dead coral colonies and coral rubble post-bleaching [145].

There were variable effects of both initial and repeated thermal stress on the increase in available reef pavement. This is probably linked to the loss of previously dominant hard and soft coral cover following the 2015–2017 marine heatwaves on these reefs. The overall gain in available reef pavement also coincides with an increase in fish community herbivory about 2 years following the warming events [146]. By sustaining high levels of grazing on endolithic and epilithic algae following bleaching, herbivores can maintain a high proportion of bare reef pavement [146,147].

### 4.3. Benthic community reorganization following thermal stress

The reorganization of coral reef benthic communities following a disturbance event depends on the dynamic interaction between the nature (scale and severity) of the disturbance event and the reef community composition [148]. Marine heatwaves have been shown to cause widespread bleaching and mortality in scleractinian corals [1,123]. This subsequently modifies the network of interactions between the wider benthic community and can cause coral-dominated reefs to shift to reefs with novel alternative configurations [1,33,40,100,149]. During the 2015–2017 thermal stress events, reefs within the Indian Ocean region that experienced high to extreme levels of bleaching [149–151] altered to configurations dominated by epilithic algal matrix (e.g. Seychelles, Aldabra atoll [140]; Kenya, Zanzibar [13]) and rubble beds (e.g. Maldives [10]).

Despite high bleaching and mortality rates during the successive marine heatwaves in the Chagos Archipelago [41], there is no evidence suggesting that changes in benthic groups are indicative of a regime shift from a hard coral-dominated community (electronic supplementary material, figures S8 and S9). This potential resilience to regime shifts following bleaching in the Chagos Archipelago may be attributed to (i) the isolation of the reefs from direct anthropogenic activities (e.g. pollution, eutrophication, sedimentation) [101]; (ii) local upwelling [121], which may reduce thermal stress and deliver nutrients required by benthic communities resulting in a less severe change in community structure following the warming events [77,152]; and (iii) high fish biomass [153,154] with strong top-down control on algae (macroalgae cover less than 7%) maintained by a high herbivore density post-bleaching in the archipelago [146], which may reduce the successional dominance of algae on the reefs post-disturbance and which will also provide vacant space for coral recruitment and recovery [155]. The Chagos Archipelago also has one of the most diverse coral communities in the Indian Ocean region, which has been associated with resilience and recovery following previous severe bleaching events in the archipelago [156] as well as the Great Barrier Reef, Moorea and Jamaica [65].

Benthic community recovery is usually assessed as the return of cover to pre-disturbance levels as well as the reassembly to similar pre-disturbance taxa relative abundances [14,148,157]. Recovery to pre-disturbance configurations can take 8–13 years [148,157]. However, as the return time between climate-induced warming events becomes shorter, the ability of coral reefs to recover to pre-bleaching levels may become compromised [1,40,43]. Despite significant bleaching and mortality of key coral species such as *Acropora*, *Pachyseris*, *Echinopora*, *Isopora* and *Galaxea* during the back-to-back bleaching events in the Chagos Archipelago [21,41,158], our data show a lack of a shift in dominance 1–2 years post-bleaching. Additionally, Lange *et al*. [159] recently indicated high recovery potential with new coral recruitment across all morphotypes (branching, massive, encrusting, tabular *Acropora*, branching *Acropora*). However, the recovery trends shown by Lange *et al*. [159] in the Chagos Archipelago only describe reefs at 8–10 m. This and several post-bleaching coral reef studies similarly focus on changes occurring shallower than 10 m [1,10,40,101,159,160], limiting our understanding of how deeper reefs respond to recurring marine heatwaves.

## 5. Conclusion

It has been suggested that reefs at greater depths may escape the effects of climate-induced bleaching events [18,19,161]. By surveying shallow reefs, we show the loss of hard and soft coral cover post-heatwaves between 15 and 25 m. While less than the observed changes at shallower 5–15 m depths, our results highlight that benthic communities down to 25 m depth can be impacted by severe heatwaves. This is supported by an increasing number of studies that show reefs at greater depths, including mesophotic reefs (30–150 m) are not immune to thermal stress with widespread evidence of bleaching impacts and changes in community composition [9,20,22,24,26,30,104]. The decreasing change in cover of benthic groups with depth highlights the importance of surveying multiple depth gradients when explaining the rate at which communities can be altered after large-scale disturbance events. The effects of initial and repeated thermal stress across depths show that even remote reefs that are protected from direct anthropogenic impacts are not resistant to the impacts of elevated seawater temperatures and subsequent changes in community dynamics and zonation patterns. Given the predicted increase in the frequency and severity of climate-driven marine heatwaves, our findings support concerns about the ability of contemporary benthic communities across the depth range of shallow coral reefs (<30 m) worldwide to resist and recover from recurrent thermal stress.

## Data Availability

Data and script can be accessed on Zenodo [162]. Electronic supplementary material is available at Figshare [163].
